# Transposable Elements in the Genome of Human Parasite *Schistosoma mansoni*: A Review

**DOI:** 10.3390/tropicalmed6030126

**Published:** 2021-07-09

**Authors:** Gisele Strieder Philippsen

**Affiliations:** Universidade Federal do Paraná, Jandaia do Sul, Dr. Maximiano Street, Parana n. 426, Brazil; gistrieder@ufpr.br

**Keywords:** transposable elements, *S. mansoni*, genome evolution

## Abstract

Transposable elements (TEs) are DNA sequences able to transpose within the host genome and, consequently, influence the dynamics of evolution in the species. Among the possible effects, TEs insertions may alter the expression and coding patterns of genes, leading to genomic innovations. Gene-duplication events, resulting from DNA segmental duplication induced by TEs transposition, constitute another important mechanism that contributes to the plasticity of genomes. This review aims to cover the current knowledge regarding TEs in the genome of the parasite *Schistosoma mansoni*, an agent of schistosomiasis—a neglected tropical disease affecting at least 250 million people worldwide. In this context, the literature concerning TEs description and TEs impact on the genomic architecture for *S. mansoni* was revisited, displaying evidence of TEs influence on schistosome speciation—mediated by bursts of transposition—and in gene-duplication events related to schistosome–host coevolution processes, as well several instances of TEs contribution into the coding sequences of genes. These findings indicate the relevant role of TEs in the evolution of the *S. mansoni* genome.

## 1. Introduction

*Schistosoma mansoni* is an agent of schistosomiasis, the third most-reported global tropical disease, affecting at least 250 million people worldwide and causing 280,000 deaths annually in 78 countries [1,2]. The mature parasite dwells in humans’ blood vessels and may persist for decades in this definitive host, indicating that this species might have evolved a sophisticated genetic system to evade the host immune system [1,3,4,5]. Schistosomiasis is related to poverty conditions, due to a lack of information, sanitation, and hygiene care [1,3,6]. As a result of repeated exposure to infectious cercariae, chronic schistosomiasis is the prevalent form in endemic regions [3], with malnutrition, anemia, compromised childhood development, and reduced intellectual-function scores the common morbidities associated with this illness [3,6]. Schistosomiasis caused by *S. mansoni* infection is endemic in sub-Saharan Africa, the Caribbean islands, Suriname, Puerto Rico, Venezuela, and Brazil [1].

Currently, praziquantel is the primary pharmacological approach for schistosomiasis treatment [1,2,3]. Studies showed that this drug is safe and efficacious [1,7], achieving a 76.7% cure and 86.3% egg-reduction rates in *S. mansoni* infections [7]. Nevertheless, the risk of emerging resistance and the lack of an efficient alternative treatment reinforce the relevance of new drugs and prophylactic vaccine development [2,6]. In this context, the understanding of the genomic basis and molecular mechanisms related to schistosome biology might improve the chances of valuable target identification and, consequently, the rational development of new therapeutic resources for schistosomiasis [3,5,8,9].

Regarding the knowledge about the genomic basis of diverse species, transposable elements (TEs) constitute a relevant issue. TEs are DNA sequences apt to transpose into a host genome within the cell [10,11], enabling them to influence the evolutionary trajectory of the species [10,12,13,14,15]. Although deleterious or neutral effects are likely [16,17,18,19], TEs mobilization may cause genomic innovations that lead to fitness increment, especially under stress conditions [12,20,21,22,23]. As a result of transposition events, genes may have their regulatory or coding sequences altered, or new genes may be established [16,17,24,25,26,27,28], with a positive effect. Due to these features, TEs have been considered a powerful and fast source of genetic variability by providing a broad spectrum of raw material on which natural selection may operate, especially in conditions of severe evolutionary pressure [12,14,21].

Considering this conjecture, this review aims to comprehensively cover studies regarding TEs in the *S. mansoni* genome. Works describing TEs families from this species were revisited, as were works investigating the possible influence of TEs on schistosome speciation, on the expansion of the micro-exon genes families—related to the parasite interaction with the host immune system—and on the coding sequences of genes. Taken together, these findings shed light on the relevance of TEs on the evolution of the *S. mansoni* genome.

## 2. Transposable Elements: General Concepts and Their Identification in *S. mansoni*

TEs were discovered by Barbara McClintock in the 1940s, who described them as “controlling elements” due to the hypothesis that they could regulate gene expression [20]. Owing to the contradictions in the then-current conjecture of genome staticity, the scientific community remained skeptical about this discovery until the 1960s, when TEs conferring antibiotic resistance in bacteria were identified [29,30]. The advancement of sequencing techniques allowed the study of diverse genomes, evidencing the occurrence of TEs in virtually all eukaryotic species [11,12,31,32]. TEs are commonly kept under control by cellular silencing mechanisms—such as small interfering RNAs, DNA methylation, or histone modifications—to prevent disadvantageous events resulting from transpositions [21,33,34,35]. Novel or stressful environmental conditions may alter epigenetic modifications leading to the activation of silent TEs, providing opportunities for genomic and phenotypic innovations that favor adaptation through natural selection [12,20,21,22,23]. This feature of TEs makes them an important driving force for adaptive genome evolution and speciation processes, so that, currently, TEs are recognized as an essential source of genomic innovations able to influence the evolutionary trajectory of species [10,12,13,14,15].

According to the transposition mechanism, TEs are grouped into two classes: retrotransposons (Class I), which transpose via an RNA-intermediate-based mechanism, and DNA transposons (Class II) that do not employ an RNA intermediate in the transposition [31,32]. For elements from the first class, a new copy is initiated by the TE sequence transcription, originating an RNA intermediate that will be reverse-transcribed in DNA by a TE-encoded reverse-transcriptase enzyme [11,31]. According to specific details of their transposition mechanism, retrotransposons are classified into different orders: Penelope-like elements (PLEs), DIRS-like elements (*Dictyostelium* intermediate repeat sequence), LTR (long terminal repeat) retrotransposons, LINEs (long interspersed nuclear elements), and SINEs (short interspersed nuclear elements); LINEs and SINEs are commonly referred to as “non-LTR retrotransposons” in the literature [11,31,32,36]. According to Wicker et al. (2007), DNA transposons can be grouped into two subclasses: Subclass 1 harbors elements that transpose by the classical ‘cut-and-paste’ mechanism, mediated by a transposase enzyme, while Subclass 2 comprises the elements that transpose by a mechanism in which only one strand is displaced. Subclass 1 encompasses the TIR and Crypton orders, while Subclass 2 includes Helitron and Maverick orders [31]. TEs are also described as autonomous or non-autonomous elements; while autonomous elements encode the specific enzymatic machinery necessary for the transposition process, non-autonomous elements do not display this feature and transpose employing the enzymes encoded by other elements [10,31,32].

Several TEs have been identified from *S. mansoni* [37,38,39,40,41,42,43,44,45,46,47,48,49]. Spotila et al. (1989) characterized the first highly repeated element for this species, designed SMα, belonging to the SINE order. Drew and Brindley (1997) identified the first protein-encoding TE from the genus *Schistosoma*. This TE family was characterized from *S. mansoni* and named SR1 (schistosome retrotransposon 1), the first CR1-like non-LTR element reported from an invertebrate taxon [38]. Still, in the context of retrotransposons in *S. mansoni*, Copeland et al. (2003) reported the first LTR element, named Boudicca, and Arkhipova et al. (2003) reported evidence of a Penelope-like occurrence for this species. Feschotte (2004) described the first DNA transposon for *S. mansoni*, named Merlin_Sm1, which belongs to the Merlin superfamily.

As a result from advances in the research regarding TEs in *S. mansoni*, diverse families of non-LTR (SR2 [39], SR3 [40], Perere [43], Perere-2, -3, -4, -5, -6, -7, -8, and -9 [47]), LTR (Saci-1, -2, -3, -4, -5, -6, and -7 [43,47], Fugitive [45], Sinbad [46], Nonaut-1.1, -3, -4.1, -5, and -6.1 [47]), Penelope-like (Perere-10 [47]), CACTA (SmTRC1 [48]), and Mutator-like (Curupira-1 and Curupira-2 [49]) groups have been fully characterized. The sequencing of the *S. mansoni* genome allowed its TE content recognition, being the non-LTR group the most representative, followed by the LTR group (15% and 5% of the genome, respectively) [50,51,52]. The authors identified a total of 72 families of LTR elements, assigned to the Bel and Ty3/Gypsy clades, and 72 families of non-LTR elements, assigned to the CR1, R2, and RTE clades [50,52]. DNA transposons have a lower representativity (less than 1% of the genome [49]) in this species when compared to the retrotransposons. Studies concerning the TEs effects in the *S. mansoni* genome are revisited in the next section.

## 3. TEs Impact on the *S. mansoni* Genome

### 3.1. Influence of Transposition Bursts in Speciation Processes

The literature has reported the potential of TEs bursts in adaptation and speciation processes [12,14,36,53,54,55]. The amplification of TEs copy number increases the raw material for genomic innovations, incrementing the genome plasticity and enabling its restructuring, which may mediate adaptation and speciation [12,14,55]. The work of Schrader and Schmitz (2019) emphasized the fact that elevated TEs activity may result in genetic diversification within populations, originating adaptive variants that might evolve and become fixed by natural selection. Belyayev (2014) explained that TEs bursts could constitute a genome reaction resulting from abrupt changes in environments. Another important mechanism of genome evolution associated with retrotransposons bursts is the establishment of retrogenes, originated by the reverse transcription of cellular mRNA, which may evolve new coding or regulatory features also influencing the speciation process [10,12,54]. A recent study concerning gene-duplication events in *S. mansoni* revealed that among the 1886 intron-less genes analyzed, 235 are putative retrogenes derived from retroposition [56], which indicates the relevance of this mechanism in the expansion of genic repertoire also in the schistosomes.

The TEs survey in the genome of *S. mansoni* and *S. japonicum* indicated that the non-LTR group is the most representative in both species, accounting for approximately 15% and 8% of the genomes, respectively [50,52,57]. Venancio et al. (2010) pointed that the significantly higher non-LTR content for *S. mansoni* when compared to *S. japonicum* indicates that this TE group was the most dynamic after the divergence of the two species. Considering this, they performed a comprehensive evaluation of the non-LTR content and found that two RTE families were expressively more common in *S. mansoni*: SR2 and Perere-3/SR3 [52] (Perere-3 and SR3 were considered a unique family by Venancio et al. (2010), designated as Perere-3/SR3, due to their high similarity in the transcriptase reverse domain). The SR2 family exhibits few and eroded copies in *S. japonicum* (corresponding to 0.10% of the genome), while in *S. mansoni*, this family displays abundant and few degraded copies (accounting for 2.94% of the genome), indicating a relatively recent burst of transposition [52]. In the case of Perere-3/SR3, several copies were identified for both species (accounting for 3.38% of the genome in *S. japonicum* and 4.84% in *S. mansoni*), evidencing that this TE family was very active in the last common ancestor [52]. Analysis of pairwise distances between nucleotide sequences from reverse transcriptase of Perere-3/SR3 copies showed a similar pattern of pairwise distance distribution for both species [52], with shorter distances more frequent for *S. mansoni*, indicating that this species was subject to a more recent expansion in the copy number of Perere-3/SR3.

The work of Venancio et al. (2010) corroborates the hypothesis that bursts of retrotransposons might have contributed to the schistosome speciation processes in Africa. A previous model proposed that African schistosomes might have originated from a migrating ancestor from Asia via mammal migration [58,59]. Considering this, Venancio et al. (2010) suggested that “the higher content of transposable elements in the *S. mansoni* genome would be a consequence of the selection of parasite populations in a new environment during the migration and speciation process of schistosomes in Africa”. The authors also pointed out that evolutionary theories predict that a population in a new territory tends to accumulate mutations [60], which might explain the selection of populations with higher TEs activity due to their property in providing raw material for genomic innovations, favoring evolutive and speciation processes [12,55,61]. 

Makałowski (2000) pointed out that TEs “should not be viewed as genomic parasites, but rather as genomic symbionts that create a *genomic scrap yard*, the source of ‘junk’ that natural selection utilizes in its evolutionary experiments”. Considering that novel environmental conditions require a rapid adaptive response that may be achieved by transposition bursts [12,55,62] and the observed relation between TEs activity and speciation processes described in the literature [14,55], the work of Venancio et al. (2010) proposed a coherent hypothesis concerning the TEs bursts influence on schistosomes speciation. In the following subsections, findings regarding TEs influence on gene-duplication events—associated with schistosome–host coevolution—and on genic architecture in *S. mansoni* will be reviewed.

### 3.2. Micro-Exon Genes Coevolution Mediated by TEs in S. mansoni

Pathogens—such as viruses, bacteria, and fungi—exhibit a relatively high speed of adaptation, stemming from interactions with host defense mechanisms [63,64]. The study of the molecular basis of pathogen adaptation in fungi revealed a relevant contribution from TEs in this regard [22]. As reviewed by Schrader and Schmitz (2019), the over-representation of genes related to host–parasite interactions in TE-rich genome regions suggests that TE-derived adaptability is a critical force in the pathogens coevolution. 

*S. mansoni* has a class of micro-exon genes (MEGs) coding for proteins exposed to the host immune system and expressed mainly in the intramammalian stage [65]. This class of genes seems to be restricted to schistosomes, since no homologous proteins were found in other genera [4,65]. The 72 MEGs, grouped into 25 families, display a particular and striking feature: they are composed mainly by short (≤36 base pairs) and symmetric exons that may be removed from the transcript without disrupting the reading frame, favoring protein variation [4,50,65,66]. DeMarco et al. (2010) proposed that the variation in the MEGs’ protein repertory mediated by the alternative splicing might provide an essential molecular mechanism to evade the host immune system in the schistosomes.

The fact that schistosomes can persist for decades in the bloodstream, despite exposure to the host immune system, indicates that they must have evolved an efficient immune evasion strategy [65,67,68]. Proteins secreted or anchored at the tegument surface, the external layer of the mature worm, are crucial players in the molecular mechanisms relative to this process [8,68,69]. All MEGs proteins carry a signal peptide and are predicted to be secreted or located in the parasite plasma membrane [50,65,66], with several MEGs being upregulated in the definitive host-invasion process [70]. Compared with other schistosomes genes, MEGs display significantly higher dN/dS values [67], indicating that they are exposed to additional selective pressure not affecting regular genes, probably imposed by the host immune system. It is expected that genes under positive selection exhibit a more significant number of nonsynonymous substitutions (dN) compared to the number of synonymous substitutions (dS), since nonsynonymous substitutions may lead to amino acid changes [71]. The evidence considered suggests that MEGs constitute a vital subject in the context of the schistosome–host coevolution process [67].

The analysis of MEGs in the *S. mansoni*, *S. japonicum*, and *S. haematobium* genomes revealed species-specific gene-duplication events [67]. Gene duplication is a suitable evolutive process because the new copy may diverge and originate innovations or specialized functions, while the original copy may conserve the initial features or also become specialized, increasing the proteome diversity [72,73]. The fact that TEs may originate gene-duplication events, through processes such as segmental duplication arising from nonallelic homologous recombination or double-strand breakages [23,61,74], instigated the analysis concerning TEs distribution in the MEGs and their environs in the *S. mansoni* genome, since the gene boundaries and a TE library were defined for this species. The results revealed an enrichment of TEs in the MEGs’ genomic regions, with a statistically significant overrepresentation (*p*-value < 0.05) for Sm, SMα, and Perere-3 elements [67]. This finding strongly suggests that *S. mansoni* genome dynamism, in terms of duplications events leading to the expansion of MEGs families, might have been accelerated by the TEs activity [67], corroborating the hypothesis that TEs might have mediated the coevolution of these schistosome-exposed proteins. This observation converges to the hypothesis present in the literature, according to which TE-derived adaptability is a fundamental factor in the coevolution of pathogens.

DeMarco et al. (2010) also described an interesting TE-related exon-duplication event in a member of the MEG-3 family. Just after the signal peptide coding sequence, the MEG-3.1 gene exhibits an additional micro-exon (9 bp length, which is identical to that found in the MEG-3.2 gene) whose environ is flanked by a TE sequence similar to the SMα retrotransposon [65]. Based on a detailed inspection of the genomic context of this duplicated exon, DeMarco et al. (2010) proposed that the repair process induced by a double-strand break originated from a TE insertion might have originated the copy of the region containing the micro-exon, leading to the exon-duplication event observed. According to the authors, this instance also indicates the TEs potential to influence the evolution of gene structures in *S. mansoni*.

### 3.3. TEs Contribution to the Evolution of S. mansoni Coding Regions

The advancement in the sequencing of several genomes has indicated TEs occurrence in coding sequences for diverse species, highlighting their relevance to the evolution of gene structures. TEs open reading frames (ORFs) can be co-opted in a new function in the host genome by a process called domestication [29,75]. Furthermore, a noncoding TE sequence can be recruited into a new function, influencing the evolution of genes through the extension of coding sequences, the insertion of alternative start or stop codons, or the introduction of alternative potential splice sites, leading to diversification in transcripts and protein isoforms [16,25,76,77].

One of the most studied cases involving transposase domestication is related to the RAG1 protein, intrinsic to the adaptive immune system in jawed vertebrates [75,78]. Evidence indicated that the RAG1 core and the V(D)J recombination signal sequences are derived from Transib, a superfamily of DNA TEs [78]. Concerning the exaptation of the TEs non-coding regions, instances were described for distinct species in the literature [25,76,79,80]. In particular, the work of Nekrutenko and Li (2001) showed that about 4% of human genes possess TE-derived sequences in coding regions, indicating the influence of these elements in the context of protein evolution in the human species.

**Figure 1:**
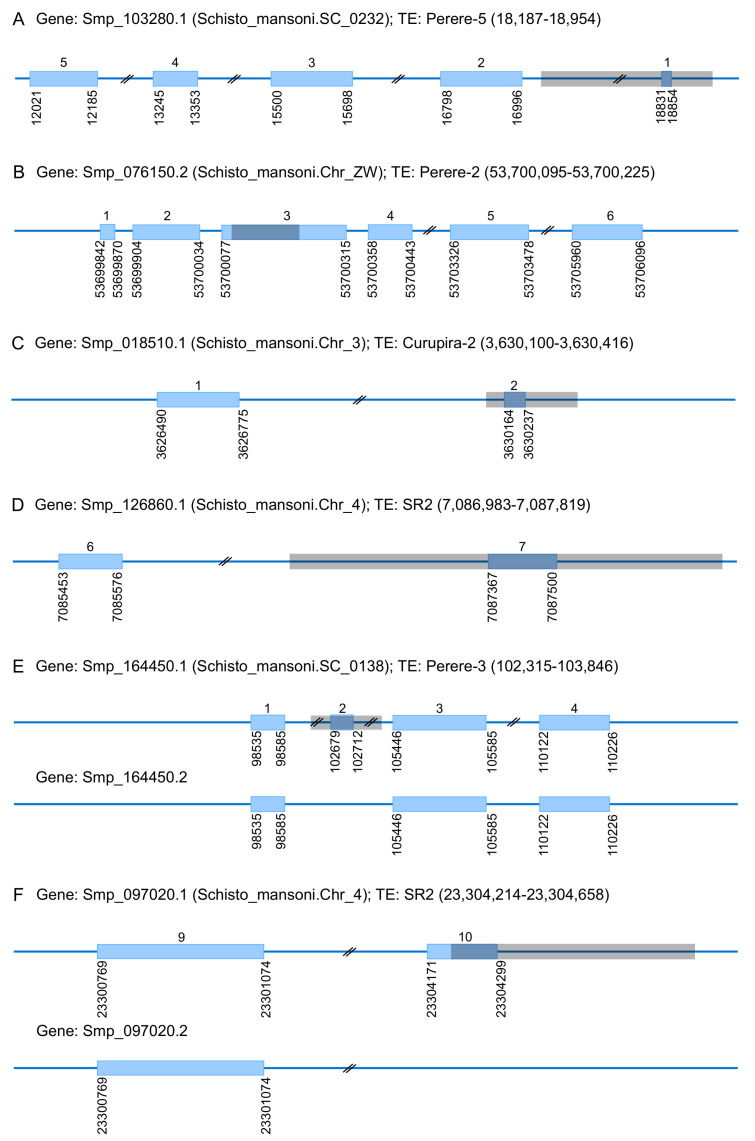
Schematic representation of the *S. mansoni* genes with a TE-derived sequence into coding regions. The blue rectangles represent the coding regions, and the gray transparent rectangles represent TE sequences. The instances pictured were previous confirmed by EST or RNAseq data [81]. Figure adapted from [81].

Considering this essential feature of TEs, the availability of the genome data [50,51], and a library of curated consensus sequences of TEs for *S. mansoni*, a detailed inspection concerning TEs impact on the coding regions for this species was performed by our group [81]. Six instances of TE occurrence in coding exons of genes (Figure 1) could be confirmed based on physical evidence of their transcription by EST or RNAseq public data [81], associated with Perere-2, Perere-3, Perere-5, Curupira-2, and SR2 elements. Interestingly, two of these six instances are related to SR2 and Perere-3, TE families that were subject to transposition bursts.

In the context of the exaptation process, TEs may become part of the genes’ coding regions through two mechanisms: by direct TE insertion into the coding DNA or by TE insertion at a noncoding DNA followed by the recruitment of this sequence as a new exon, due to the potential splice sites existing in the TEs [25,82]. Among the six genes in *S. mansoni* that have been inspected in detail [81], four exhibit an entire exon derived from a TE (Figure 1A,C–E), evidencing the TEs potential to shape the architecture of genes in this species.

Still concerning the six instances identified in *S. mansoni* [81], it was possible to observe alternative transcripts to Smp_164450.1 (Figure 1E) and Smp_097020.1 (Figure 1F) genes resulting from the TE-derived exon skipping. These constitute interesting cases of a proteome repertory increasing through alternative splicing related to TEs in *S. mansoni*, demanding further studies to the complete understanding of their specific biological role. The alternative splicing is a post-transcriptional step that occurs in different cell types and in different developmental conditions, tending to be more frequent in more complex organisms [73]. This mechanism has been recognized as a major player in improving transcriptome and proteome repertory [73,83,84], especially in mammals, being the TEs recognized as a source of innovations in this regard due to the potential splice sites carried by them [25,77]. 

## 4. Conclusions

The advancement in sequencing techniques has allowed the recognition of the TEs essential role in the genome evolution of diverse species. This review shed light on studies regarding TEs description and content in the *S. mansoni* genome, as studies displaying evidence of TEs influence on schistosome speciation. The fact that MEGs are located in TE-rich genomic regions suggests that TEs activity might have accelerated duplication events identified for these genes. Considering that MEGs proteins are related to parasite interaction with the host immune system, TEs might have influenced the schistosome–host coevolution process by inducing the expansion of the MEGs families, improving the protein repertory and the pathogenicity associated with the parasite. The instances of TE-derived sequences in the coding regions of *S. mansoni* genes were also observed, attesting to the TEs potential in shaping the genic architecture. The findings described in the revisited works provide strong evidence that TEs were pivotal in establishing genomic innovations in *S. mansoni*, acquiescing plasticity and adaptability for the genome evolution in this species. Future studies are required to fully understand the impact of TEs heritage on the biology of *S. mansoni*. All reports reviewed corroborate the view of TEs as a substantial driving force of genome evolution, also in the genus *Schistosoma*.
